# Experiences of Accessing and Providing Contraceptive Implant Removal Services in Gaborone, Botswana: A Qualitative Study Among Implant Users and Healthcare Providers

**DOI:** 10.3389/fgwh.2021.684694

**Published:** 2021-06-25

**Authors:** Rebecca Howett, Emily A. Krogstad, Opelo Badubi, Alida M. Gertz, Caitlin Bawn, Aamirah Mussa, Tiroyaone Kgaswanyane, Sifelani Malima, Tshego Maotwe, Lesego Mokganya, Doreen Ramogola-Masire, Chelsea Morroni

**Affiliations:** ^1^Botswana-UPenn Partnership, Gaborone, Botswana; ^2^Desmond Tutu HIV Centre, University of Cape Town, Cape Town, South Africa; ^3^Botswana Harvard AIDS Institute Partnership, Gaborone, Botswana; ^4^Sexual and Reproductive Health Department, Elizabeth Garrett Anderson Institute for Women's Health, University College London, London, United Kingdom; ^5^Research Department of Primary Care and Population Health, University College London, London, United Kingdom; ^6^Botswana Family Welfare Association, Gaborone, Botswana; ^7^Botswana Training and Education Center for Health, Gaborone, Botswana; ^8^Botswana Ministry of Health and Wellness, Gaborone, Botswana; ^9^Department of Obstetrics and Gynecology, University of Botswana, Gaborone, Botswana; ^10^Liverpool School of Tropical Medicine, Liverpool, United Kingdom; ^11^Medical Research Council Centre for Reproductive Health, University of Edinburgh, Edinburgh, United Kingdom

**Keywords:** contraceptive implant, implant removal, long-acting reversible contraception, botswana, qualitative study

## Abstract

**Introduction:** This study explored implant user and healthcare provider experiences of accessing and providing contraceptive implant removal services in Gaborone, Botswana, following introduction of the implant in the public sector in 2016. We sought to understand reasons for satisfaction and dissatisfaction with services and their potential impact on wider perceptions of the implant, including influence on future uptake.

**Methods:** Qualitative data were collected through in-depth interviews. Participants comprised ten women who had previously undergone implant removal, and ten providers whose work included provision of implant insertion and removal. Data were analyzed using thematic content analysis.

**Results:** Seven of the ten users in this study had experienced a delay between initial request and undergoing implant removal. This interval ranged from <1 week to 3 months. Users identified the principal barriers to accessing implant removal services as lack of access to trained removal providers, inconvenient appointment times, and provider resistance to performing removal. Nine of the ten providers in this study had experienced barriers to providing implant removal, including insufficient training, lack of equipment, lack of time, and lack of a referral pathway for difficult removals. Despite experiencing barriers in accessing removal, users' perceptions of the implant remained generally positive. Providers were concerned that ongoing negative user experiences of removal services would damage wider perceptions of the implant.

**Conclusion:** Introduction of the contraceptive implant in Botswana has been an important strategy in increasing contraceptive choice. Following an initial focus on provision of insertion services, the development of comparable, accessible removal services is critical to ensuring that the implant remains a desirable contraceptive option and is vital to upholding women's reproductive health rights. The experiences of users and providers in this study can inform the ongoing development of services for implant insertion and removal in Botswana and other lower-resource settings.

## Introduction

The subdermal contraceptive implant is a convenient and highly effective (1, 2) long-acting reversible method of contraception (LARC) which is medically safe for most women (3). Over the past decade, there has been a global increase in implant use (4) and uptake has been particularly marked in sub-Saharan Africa (5). The Botswana Ministry of Health and Wellness (MoHW) established a public sector implant program in 2016 (6), providing both a one-rod etonogestrel implant (Implanon NXT®, Merck), and a two-rod levonorgestrel implant (Jadelle®, Bayer).

Increasing implant demand has created a corresponding demand for implant services, both for initiation and for discontinuation, as the method is dependent on access to trained healthcare providers for insertion and removal. The implant has a lifespan of 3–5 years of contraceptive effectiveness, depending on model, after which it should be replaced. Users may also opt to discontinue before its full lifespan has elapsed due to problematic side effects (most commonly heavy or irregular vaginal bleeding) and desire to conceive (7–10).

In order to uphold women's reproductive rights, they should be empowered to exercise full contraceptive autonomy (11), which includes the right to discontinue any given contraceptive method at their choosing (12). Thus, access to removal services is essential from the moment of implant introduction. Unfortunately, in many settings upscaling of removal services has not always kept pace with that of insertion services (4). Perception and subsequent uptake of the implant have previously been damaged by perceived difficulty in accessing removal services, including with Norplant® (Wyeth-Ayerst), a six-rod levonorgestrel predecessor to the current implant models, in the 1990s (13) and more recently with Implanon NXT® in South Africa (14).

We understand the reasons why women request implant removal, but there is a lack of information on women's experiences with accessing removal services. This information is particularly sparse in resource-limited settings where healthcare systems face multiple concurrent challenges. We aimed to gain an understanding of experiences in accessing and providing contraceptive implant removal services. We explored this issue from both sides: implant users who have sought removal, and healthcare providers who have experience in removing implants. We sought to identify reasons for satisfaction and dissatisfaction with the process, and to determine whether challenges encountered in accessing implant removals have had an impact upon wider perceptions of this method of contraception, including any influence on future uptake. This research has relevance for the provision of implant insertion and removal services in Botswana, as well as internationally, and thereby can contribute to upholding women's reproductive rights.

## Methods

### Study Setting

This study was conducted in Gaborone, Botswana's capital city and largest urban center, between April 2018 and July 2018. About 11% of Botswana's total population resides in Gaborone, and 64% of Botswana's total population resides in urban areas (15). Women who had previously used and then discontinued the implant (“users”) were interviewed at a non-governmental organization (NGO) sexual and reproductive health (SRH) clinic but had received implant services in both government and NGO clinics. The NGO clinic provides SRH services to residents of Gaborone and its surrounding settlements, and also acts as a referral center for other local clinics. Healthcare providers (“providers”) were interviewed at their place of work.

### Study Design

Qualitative data were collected through in-depth individual interviews using interview guides which were developed for this study. These guides were pre-piloted with women and providers who were not participants. The interview guide consisted of a brief introduction to the purpose of the study, followed by questions on socio-demographics, reproductive/contraceptive history (for users) or professional background (for providers). Users were asked about their decision-making surrounding initiation and discontinuation of the implant. Both groups were asked about their experiences with implant removal services, and their overall experiences and perceptions of the implant as a method of contraception. Ethical approval was obtained from the Botswana Health Research and Development Committee, the University of Botswana Research Ethics Committee, and University College London (UK) Research Ethics Committee.

### Study Participants and Recruitment

Eligible users were women aged 18 years or older who had undergone implant removal in the Botswana public sector health services or an NGO clinic within the past 3 months (we unintentionally interviewed one participant who had undergone implant removal within the past 6 months but still included this user in the analysis). We did not specify early removal of the implant (i.e., before its full lifespan had elapsed) amongst the inclusion criteria; however, all users in our study had discontinued early. Users were identified opportunistically when they presented to the NGO clinic for any reason.

Eligible providers had undergone implant training within the Botswana implant program, and currently work in provision of contraceptive services including implant insertion and removal. Providers were identified from the Botswana MoHW list of implant providers. A sample size of approximately ten users and five providers was defined in the study protocol, but we recruited until the point of data saturation.

All potential participants who were approached consented to be interviewed.

### Data Collection

Interviews were arranged at a time and place convenient for each participant. Written informed consent was obtained prior to participation. Interviews were conducted in the participant's preferred language (Setswana or English), in a private room, and with only the interviewer and the participant present. All interviews were digitally audio-recorded and anonymized. User interviews were conducted by author OB (a female research assistant). Provider interviews were conducted by authors AMG (a female research fellow) and CB (a female doctoral student). All three interviewers are experienced in conducting SRH research and hold relevant master's level qualifications, AMG also holds a medical degree and is experienced in providing clinical care in this research setting. Users were compensated 50 Botswana Pula (≈ 4 USD); providers did not receive any compensation.

### Data Management and Analysis

Interviews were transcribed by OB and RH, with translation of Setswana into English by OB. Transcripts were analyzed using Dedoose web-based software (SocioCultural Research Consultants, LLC, Los Angeles, USA). Demographic data were collected on a REDCap (Research Electronic Data Capture) database. Interview transcripts were analyzed using thematic content analysis (16) by a team of three research members (RH, AMG and OB). An initial codebook of codes and sub-codes was developed based on emerging themes in the user interviews. The codebook was revised through review of field notes and discussion with the analysis team, and was further revised following completion of the provider interviews. Some codes were developed as being relevant to themes expressed by both users and providers, and other codes were developed as being specific to one participant group. Please see Supplementary Tables 1, 2 for a summary of key codes. Initial coding was done by RH, then codes and associated transcript excerpts were reviewed by AMG and OB for relevance and discussion. After coding, further analysis was completed using a matrix framework to aid grouping of themes. Interpretation of linkages and meaning was discussed by the analysis team, and a consensus was reached on the key themes. All discussions took place via telephonic consultations.

## Results

### User Characteristics and History of Accessing Implant Services

In order to reach data saturation, ten in-depth interviews were conducted with women who had previously undergone removal of the contraceptive implant. User interviews were in English (*n* = 1), Setswana (*n* = 4), or a mixture of English and Setswana (*n* = 5), with a mean duration of 38 min.

User characteristics are presented in Table 1. Median age of users was 25.5 years (range 22–38 years). One user had used Jadelle® and nine had used Implanon NXT®. Insertions had been performed at six different healthcare facilities: three government clinics in Gaborone (*n* = 5); one NGO clinic in Gaborone (*n* = 3); one government clinic in an outlying district (*n* = 1); and one government hospital in an outlying district (*n* = 1). The median interval between implant insertion and removal was 137 days (range 68–679 days). Primary reasons for removal were side effects (*n* = 9, principally amenorrhoea, irregular vaginal bleeding, headache and weight gain) and desire to conceive (*n* = 1).

**Table 1:** User characteristics and history of accessing implant services.

**Characteristic**	***n* = 10**
Age (years)	25.5 (22–38)
**Employment status**	
Employed	2 (20%)
Student	3 (30%)
Unemployed	5 (50%)
**Highest level of education**	
Junior secondary	1 (10%)
Senior secondary	1 (10%)
Tertiary	8 (80%)
**Resident in gaborone**	
Yes	9 (90%)
No	1 (10%)
**HIV status**	
Positive	0 (0%)
Negative	10 (100%)
**Current partnership status**	
Single	1 (10%)
In a relationship	8 (80%)
Married	1 (10%)
**Number of pregnancies**	
0	3 (30%)
1	5 (50%)
≥2	2 (20%)
**Number of children**	
0	3 (30%)
1	6 (60%)
≥2	1 (10%)
Age at first pregnancy (years)	21 (17–22)
Age at most recent pregnancy (years)	22 (19–29)
**Type of implant used**	
Implanon NXT®	9 (90%)
Jadelle®	1 (10%)
**Primary reason for removal**	
Side effects	9 (90%)
Desire to conceive	1 (10%)
**Number of clinics attended for insertion**	
1	9 (90%)
>1	1 (10%)
**Number of clinics attended for removal**	
1	6 (60%)
2	2 (20%)
3	1 (10%)
4	1 (10%)
**Number of clinic visits for removal**	
1	3 (30%)
2	5 (50%)
3	1 (10%)
≥4	1 (10%)
**Delay to removal**	
None	3 (30%)
<1 week	1 (10%)
1–2 weeks	4 (40%)
3 months	2 (20%)
**Interval between insertion and removal (days)**	137 (68–679)
**Other methods of contraception ever used**	
Copper IUD	1 (10%)
DMPA injection	5 (50%)
Oral contraceptive pill	4 (40%)
Male condom	9 (90%)
Withdrawal	1 (10%)

*All data are presented as n (%) or median (range); HIV, human immunodeficiency virus; IUD, intrauterine device; DMPA, depomedroxyprogesterone acetate*.

Seven of the ten users had made two or more clinic visits while seeking implant removal, and for four users these visits had been made to two or more different clinics. The approximate interval from initial request to undergoing removal ranged from the same day to 3 months. Removals had almost exclusively been performed at the NGO clinic, with one exception: one user who had used a Jadelle® implant had undergone two removal procedures; one rod had been removed at a government clinic in Gaborone, and the second rod was removed at the NGO clinic after referral.

Following implant removal, none of the users had chosen to use the implant again by the time of interview. Three users switched to the male condom, two switched to the oral contraceptive pill, one switched to the DMPA (depomedroxyprogesterone acetate) injection, and one was relying on withdrawal. Three users did not restart any method of contraception. This was due to desire to conceive (*n* = 1), current sexual abstinence (*n* = 1), and wanting to “*recover from all the hormones”* (*n* = 1). Two of the three users who switched to condoms also stated that they wanted to avoid hormonal methods of contraception.

### Provider Characteristics and Professional Experience

Ten in-depth interviews were conducted with providers who had experience in providing implant services. All provider interviews were in English, with a mean duration of 58 min.

Provider characteristics are presented in Table 2. Nine providers were female, and one was male. Providers were based at eight different healthcare facilities in the Gaborone area: one NGO clinic; six government clinics; and the gynecology department of a main government hospital. All providers had completed nursing training, eight had undergone additional midwifery training, and two had undergone additional implant training to become Master Trainers in the Botswana implant program. Six providers had been trained on Implanon NXT®, two had been trained on Jadelle®, and two (the Master Trainers) had been trained on both types of implant. All providers had undertaken their original implant training through the Botswana implant program between July 2016 and October 2017, and six had undertaken refresher training. The median number of insertions per provider was 125 (range 40–400) and the median number of removals was 15 (range 0–100). One provider had not performed any removals due to no demand at her clinic.

**Table 2:** Provider characteristics and professional experience.

**Characteristic**	***n* = 10**
Total clinical experience (years)	16 (6–23)
Duration in current post (years)	3 (1.5–8)
**Type of implant covered in training**	
Implanon NXT®	6 (60%)
Jadelle®	2 (20%)
Both	2 (20%)
Interval from original training to interview (months)^*^	23 (8–27)
**Has undergone refresher training**	
Yes	6 (60%)
No	4 (40%)
Estimated number of insertions performed	125 (40–400)
Estimated number of removals performed	15 (0–100)

* *Data were only available for 9 providers*.

*All data are presented as n (%) or median (range)*.

### Barriers Experienced in Accessing Implant Removal Services

Three users reported no barriers to accessing implant removal services and had undergone this procedure on the day of their first clinic presentation for removal. The other seven users had all experienced delays. The principal reasons for delays were lack of access to trained removal providers, inconvenient appointment times, and provider resistance to remove the implant.

For many users, delay in accessing implant removal was due to no trained removal provider being available at their local clinic leading to repeat visits to multiple clinics. In some cases, users made an appointment at the first visit and later returned to the same clinic on a day when a removal provider was available. In other cases, no removal provider was working at the first clinic so users visited other clinics in search of a trained provider. Users questioned the underlying programmatic setup which led to such circumstances: “*I failed to understand why a clinic that does implant insertions could not have personnel that remove it. It does not make sense to me”* (User 02, age 35).

Even when a removal provider was available, users highlighted inconvenient appointment times as an additional barrier to accessing implant removal. This led to further delays, and several users had missed school or work to attend the clinic.

Judgmental provider attitudes or perception of providers as being resistant to users discontinuing the implant was a recurrent theme in user interviews. Interactions of this nature became a negative part of users' implant experiences. One user explained that, “*I personally really liked the implant. It is just that when it comes to removing it, healthcare providers become really slow and reluctant in removing”* (User 05, age 38). In some cases, users perceived resistance from providers who were not able to perform removals: “*I first went to [a government clinic], and when I got there and told them I want to remove the implant, they said ‘What? We do not do that here,' with that attitude”* (User 02, age 35). In other cases, providers had undergone implant removal training but attempted to persuade users to continue using the implant until its expiry date. One user described how “*[The provider] was advising me against it ‘no, just finish off, you are not left with much time”'* (User 08, age 28). These descriptions were corroborated by providers who had heard of colleagues expressing negative attitudes toward patients: “*They [users] will say, ‘they [providers] were not friendly', so they will be going from one clinic to another, to another”* (Provider 07, government clinic).

The narratives of two users, whose experiences illustrate these themes, are summarized in Table 3 as Narratives A and B.

**Table 3:** Illustrative narratives of user experiences accessing implant removals services.

**Narrative A:** User 01 is aged 25 years and has one child. She used an Implanon NXT^®^ implant for 15 months but decided to remove it due to associated side effects. She had experienced recurrent headaches, tiredness and weight gain since initiating the implant. She also disliked the loss of her usual menstrual cycle, *“I was not getting my period, I just felt dirty.”* She felt that providers failed to address her concerns, *“I just feel that I was not listened to, both the two nurses did not listen to me.”* She attended three different clinics seeking implant removal, *“I have been going to clinics for months now looking to remove it.”* She encountered provider resistance at the first clinic, *“I kept being postponed… [The provider] said that she doesn't know what it is with women, they come running to insert the implant and then they are the very same people who come running to have it removed.”* At the second clinic, she could not get an appointment because there was only one implant provider, *“When I got there the lady would always yell and put down dates for me that were very inconvenient as I was still working at the time.”* She underwent implant removal at the third clinic, the NGO clinic, after a total delay of 3 months.
**Narrative B:** User 05 is aged 38 years, has three children and lives in a village to the west of Gaborone. She used a Jadelle® implant for 5 months after having it inserted at a government clinic in her neighboring village. She disliked side effects, including lack of menstrual bleeding, and also experienced abdominal pain. First, she went to the clinic where she had the implant inserted, “*where I was given the runaround and they just refused and I gave up.”* This clinic encouraged her to persist with the implant and advised that it might not be the cause of her symptoms. Then she went to a government clinic in a neighboring town, “*where I went several times until I gave up.”* She then traveled 40 km to a government clinic in Gaborone, where she encountered further resistance from the providers and made multiple visits to request implant removal. “*After going there several times, and only upon realizing that I meant business about removing, the nurse actually removed one [rod] and then she tried removing the second [rod] but realized that she could not because it was too deep.”* The nurse referred her to the NGO clinic where the second rod was safely removed. Overall she visited four clinics, two of which on multiple occasions, and had a delay of 3 months from initial request to obtaining implant removal.
**Narrative C:** User 06 is aged 23 years and has one child. She usually goes to the NGO clinic for family planning services, and this is where she had an Implanon NXT® implant inserted. She had the implant removed at the same clinic two and a half months later due to side effects (headache, irregular vaginal bleeding). She had no delay to removal, having had it removed at her first clinic visit requesting removal (“*It was very easy, they didn't give me any grief”*).

*NGO, non-governmental organization*.

### Barriers Experienced in Providing Implant Removal Services

Providers described the principal barriers to providing implant removal services as insufficient training, lack of equipment, lack of time, and lack of a referral pathway for difficult removals. Only one provider stated that they had experienced no barriers to providing removals.

In common with users, providers reported there was a lack of trained removal providers. They described being the only trained provider at their clinic and not always being available to perform implant removals: “*I'm alone here: I'm the one who's doing the insertions, I'm the one who's doing the removals. And I'm working on shifts. Sometimes they come here and I'm not here. I'm off or I'm on night duty”* (Provider 10, government clinic); “*Patients come here, they don*'*t find me, they get frustrated”* (Provider 06, government clinic).

In addition to a lack of fellow providers, they also felt that implant training had been deficient in some aspects. Not all providers felt competent to provide insertions and removals following basic training, but they felt that subsequent MoHW mentoring and refresher training had addressed these concerns. Most providers described insufficient focus on removals in basic implant training, including insufficient practical experience: “*The theory part was sufficient but the practical part was not so sufficient”* (Provider 01, NGO clinic). Another issue was insufficient training on removal of the two-rod Jadelle® vs. training on removal of the one-rod Implanon NXT®: “*I still struggle with removing some implants, especially the Jadelle*^®^*, because the rods… slip around and they run around when you try… It is really easy to remove the Implanon NXT*^®^ *but the Jadelle*^®^ *still gives me a headache.”* (Provider 06, government clinic); “*When I came to practice, I could see that I am having challenges with the removal of certain implants… it takes me less than a minute to remove the Implanon [NXT]*^®^*, but when it comes to Jadelle*^®^ *it is a challenge because it is more flexible and sometimes when it is inserted, maybe it is not in the V like it is supposed to be”* (Provider 07, government clinic).

Most providers said that they lacked some of the key equipment for providing implant removals, particularly gloves and mosquito forceps which are in short supply and are also required for the provision of other services such as infant circumcision.

Provision of other services also requires providers' time, leading them to feel like they have insufficient time to perform implant removals in the face of competing demands and with “*so many clients queuing”* (Provider 07, government clinic).

Difficult removals encompass more challenging removals of implants which are not easily palpable in the usual location due to incorrect insertion or subsequent migration. Safe removal of this subset of implants requires additional expertise or equipment. Providers felt that they had undergone insufficient training on difficult removals: “*Training was sufficient for correctly inserted implants, and not necessarily for difficult removals”* (Provider 01, NGO clinic). Most providers recalled being taught not to attempt difficult removals: “*I think if I have got a difficult one, I will phone one of the doctors and I will refer. Because it has been stated clearly: if you realize the implant is deep, do not even try it, that's the thing”* (Provider 10, government clinic). Most providers were able to identify one or two destinations to which they would refer patients for difficult implant removals. This included the NGO clinic (*n* = 6), a main government hospital (*n* = 2), directly to the lead SRH consultant (*n* = 2), or that they would seek advice from the MoHW family planning team (*n* = 1). One provider at the NGO clinic did not have a referral pathway beyond their own clinic.

In addition to referring users to other providers for difficult removals, providers also overcome some of the barriers to standard removals by referring users to alternative providers. Referrals enable women to access services, even if their local provider is not available: “*Instead of just tossing patients here and there, we tell them where to get the services”* (Provider 09, government clinic).

Providers' narratives offered an explanation for their perceived resistance to performing removals, which had emerged as a recurrent theme in the user interviews. Providers described a conflict between promoting the rights of individual women to discontinue the implant, against their perceptions of the financial impact of early removal on the national family planning program. Some providers identified the cost of the implant as a factor which would dissuade them from removing it before its expiration date: “*But this thing is expensive, you think, ‘Ah, no, how could you?' She inserted in March and she removed last week. Three months. The Jadelle*®*, five year one”* (Provider 06, government clinic).

### Impact of Removal Services Experiences on User Perceptions of the Implant

User perceptions of the implant were influenced by the whole experience from counseling and initiation, to implant use, and eventual discontinuation. Our study focused on this final step: accessing services and undergoing removal for implant discontinuation.

Most users had encountered barriers to accessing removal services, as described above. Three users, all of whom had presented directly to the NGO clinic for implant removal, had not encountered any barriers to accessing these services. An example of one user who described a positive experience of accessing implant removal services is summarized as Narrative C in Table 3.

Before actually undergoing removal, many users were fearful of the invasiveness of the removal procedure: “*I was just afraid of being cut open”* (User 04, age 24). Half of users felt that they had been counseled insufficiently, or not at all, about the removal procedure at the time of implant insertion. Lack of information stoked anxiety about undergoing removal: “*I was really scared because I didn't know what was going to happen. I wasn't comfortable because I didn't know how it is removed”* (User 06, age 23). However, other users praised the counseling they had received and consequently felt less anxious: “*I understood, they were really honest about it”* (User 08, age 28). In contrast to users' mixed reports of information received during counseling, all ten providers said that they included information about removal as part of their usual pre-insertion counseling. However, they tended to focus on possible reasons for removal (with an emphasis on side effects) and where to access removal services, rather than the nature of the procedure itself.

All users interviewed were pleased with the quality of the removal procedure they had undergone and had confidence in the skills of their removal providers. Users' comments about removal included: “*It wasn't even painful”* (User 01, age 25); “*They were very nice. I was in good hands”* (User 04, age 24); “*It didn't take as long as I thought it would. I think they handled it well”* (User 07, age 26).

Users perceived that the positive aspects of their implant experience (reliable and convenient long-acting contraception) outweighed the negative aspects (side effects, difficulty in obtaining removal): “*It works and does what it is supposed to do which is prevent pregnancy, so really, I respect it”* (User 04, age 24), “*When the good surpasses the bad then everything is good”* (User 08, age 28). Users' assessment of the implant being overall advantageous led the majority to say that they would consider using it again in the future. Most users cited an improvement in side effect profile as being the factor which would most attract them to choosing the implant again. For many users, their experience of side effects had been more negative than their experience of using removal services. All users said that they would recommend the implant to other women. Users wanted to improve access to services for initiating the implant so that potential users could follow up on their recommendation: “*I wish [the implant] could be easily accessible and available everywhere. With me, I managed to get it because I was persistent… You [should not] have to move from clinic to clinic looking for [implant insertion services]”* (User 02, age 35).

Users reasoned that others might not experience the same side effects and that they could benefit from the positive attributes of the implant. For example: “*It really is a good method and it's effective. Just that I was unlucky it didn't agree with me, but it's a good method”* (User 03, age 22); “*People are different. The implant might be good for someone but it can also be not good for other people, but I would recommend that people use it”* (User 06, age 23). In addition to the previously well-documented side effect of heavy or prolonged vaginal bleeding leading to implant discontinuation, participants in our study also identified lack of bleeding (i.e., oligo- or amenorrhoea) as a reason for early implant discontinuation. Users believed menstruation to be associated with bodily cleansing and therefore associated lack of regular menstruation with uncleanliness: “*I just did not like that I wasn't cleansing monthly”* (User 09, age 27).

Overall, despite encountering barriers in accessing removal, users' perceptions of the implant remained positive. Perceptions had been damaged to a greater degree by issues with side effects than they had by the difficulties experienced in accessing removal services.

### Impact of Removal Services Experiences on Providers' Perceptions of the Implant

In contrast to the perceptions of users, providers expressed greater concern that ongoing negative experiences of removal services would lead to user dissatisfaction and endanger the overall image of the implant: “*I strongly believe it is going to affect the insertion rate of implant. Because if they share that they wanted to remove it but they couldn't, I don't think other people are going to want to insert. It is frustrating”* (Provider 06, government clinic). Other providers thought that women's current experiences might generate negativity toward individual clinics or providers, rather than toward the method as a whole: “*I don't think that gives them a bad perception towards the implant as a method, rather it will be towards us as providers”* (Provider 02, NGO clinic).

Providers agreed with users in calling for increased access to implant insertion services. Providers overwhelmingly thought that the implant was a suitable method of contraception for Botswana and that it should be promoted widely, despite existing barriers with removal services.

### Recommendations for Improving Access to Implant Removal Services

Participants from both groups suggested that access to removal services could be improved by strengthening provider training. In addition to training more providers, participants also called for improved training of existing providers in relation to the whole implant user journey: pre-insertion counseling, insertion, counseling during use, and removal.

Providers perceived that poor insertion techniques, particularly deep rod placement, led to subsequent difficult removals. They lacked confidence in technical skills for performing implant removals following initial implant training. They spoke positively about subsequent in-service training which, they reported, was effective in improving their skills and competency. Providers also called for more practical sessions in implant training, and ongoing in-service training and mentoring to improve standards in both insertion and removal.

Users and providers consistently recommended that better counseling should be provided to improve experiences with implant services in the future. Half of users felt that they had received insufficient pre-insertion counseling about the eventual removal procedure, despite all ten providers saying that they routinely counsel potential implant users about discontinuation and removal. One provider thought that improved pre-insertion counseling on possible side effects would lead to fewer users choosing early implant removal: “*You find that in most cases there has been insufficient counseling prior to the insertion because when you do the further counseling, the clients then decide to keep the implant”* (Provider 01, NGO clinic). The same theme was expressed in one of the user interviews: “*So because the nurse explained [possible side effects] to me I wasn't that worried. Because they told us that others you would spot [experience irregular light vaginal bleeding] every day, whereas with others the period would diminish”* (User 08, age 28).

Many users had encountered provider resistance to early removal of implants before their expiration dates. One provider emphasized the importance of upholding each woman's right to discontinue the implant when they choose to and thought that fellow providers should have increased training on facilitating users' choice: “*We should focus a lot on… talking to the service providers about the clients' rights to remove the implant when they want to… Sometimes [providers] refuse to remove [the implant], citing things like ‘it's expensive' and ‘you have just recently inserted it.' If the client is uncomfortable then they are free to remove [the implant]. We should put more emphasis on that”* (Provider 01, NGO clinic).

Users and providers highlighted the need to address inequalities in access to SRH services, including the implant, for women living in rural areas: “*[In the] remotest areas, they really don't know anything about [the implant], they really don't. Yes. And also, because the healthcare providers, they don't know about the methods, they don't give information about it”* (Provider 09, government clinic); “*Go out there and teach women. And take the services to women in rural areas”* (User 04, age 24).

## Discussion

This study addresses an important aspect of contraceptive implant provision: implant removal. We sought to gain an understanding of experiences with implant removal from the perspective of both users and providers of implant services in the resource-limited setting of Botswana. Existing studies have investigated overall perceptions of the implant, reasons for early implant removal, and in a few cases, experiences of accessing implant removal services (17, 18).

Our study offers insight into the existing barriers and how these might be addressed to improve access to implant removal services. Across both participant groups, the principal barriers to accessing implant removal services were identified as: lack of trained removal providers and therefore lack of provider time; lack of equipment; lack of a referral pathway for difficult removals; inconvenient appointment times and provider resistance to performing early removals. These barriers are interrelated and can be classified under two broad areas: human resources and supply chain management.

In terms of human resources, lack of trained removal providers leads to lack of time on the part of providers who have been trained, which in turn leads to limited and sporadic provider availability such that women seeking implant removal have to make multiple clinic visits or attend at inconvenient times. In our study, many providers who had undergone basic implant training described a lack of confidence in performing removals independently in their clinics, effectively subtracting themselves from the number of removal providers which would have been expected, based on the number trained. This finding is consistent with providers' experiences following implant training in neighboring South Africa (19).

Even with improved provider training for standard implant removals, additional training and/or improved referral pathways will be required to address difficult implant removals for a small subset of users. One study of Implanon NXT® found that 13.7 per 1,000 removal procedures were classified as difficult (20). There is a lack of quantitative data on difficult removal rates for Jadelle®, nor comparison of rates of difficult removals between Implanon NXT® and Jadelle®. Specialist services for difficult implant removals have been established in other settings, including elsewhere in sub-Saharan Africa (21). Providers in our study identified where they would refer users for difficult implant removals, and for six providers, this destination was the NGO clinic. All users in this study underwent removal at the NGO clinic, but only one would likely have been classified as a difficult removal. This suggests that the NGO clinic is frequently being used for standard removals through provider referrals and user self-presentations, which could reduce its capacity to provide services for difficult removals.

Providers in this study perceived Jadelle® as being technically more challenging to remove than Implanon NXT®. Removal of Jadelle® can be facilitated by using the modified vasectomy clamp technique (22), but providers rarely have access to this equipment in Botswana. Additionally, six of the ten providers in this study did not receive specific training on Jadelle®, which may have affected their perceptions of the difficulty of the removal process. In South Africa's Western Cape Province, all Jadelle® implants are considered difficult to remove by default, and women are referred to a specialist difficult removals clinic (22). However, Jadelle® is not provided in the South African public health sector, whereas it is provided in the Botswana public health sector so we might reasonably expect implant providers to be able to remove as well as insert this implant model. Results from this study indicate that more training on how to remove Jadelle® is needed so that providers in Botswana are confident in providing removal services for both types of implants.

This study also suggested that there is insufficient supply chain management, evidenced by a lack of equipment and disposable commodities. In some cases, even if the human resource challenges had been met and a trained, competent provider was available at a time and location accessible and convenient to users requesting the service, supply issues could still thwart provision of implant removals.

Botswana has addressed human resource and supply chain challenges to successfully upscale provision of implant insertion services, but our findings suggest that further work is needed in upscaling provision of removal services. In the early days after implant introduction in Botswana, and in common with the experience of SRH services elsewhere (4), demand for insertions was higher than demand for removals and the focus was on insertion services. However, over the course of her reproductive life, each woman who uses the implant will likely undergo the same number of removals as insertions. Therefore, upscaling of implant removal services needs to be given sufficient attention in order to match the pace set by implant insertion services. All users in our study accessed implant removal at the NGO clinic, and the majority of providers identified this clinic as the healthcare facility to which they would refer patients for implant removal. Use of the NGO clinic as the main removal center might not be sustainable as a long-term strategy for provision of removal services. In other countries, integration between public sector health services and NGOs has strengthened capacity for provision of implant services, including those for implant removals. Examples of NGO and public sector collaboration for implant service provision include a Population Services International program for LARC initiation in Zambia (23) (though the description of this program does not specify services for LARC discontinuation i.e., implant removal), as well as the work of Marie Stopes International in provision of implant services in multiple countries across sub-Saharan Africa (24).

The principle of reproductive autonomy should be fundamental to the provision of all contraceptive services (25) and is key to the interpretation of our study findings. Women's reproductive rights are supported through full, free and informed choice about contraceptive options, and relate not only to facilitating the decision to start contraception, but also the decision to stop contraception (11, 26). Full choice is facilitated through the availability of accessible services for the initiation of a wide range of contraceptive methods, but also through the availability of accessible services for discontinuation of these methods. Free choice depends on women being able to voluntarily choose to use or not use contraception without barriers or coercion. Informed choice requires that women are provided with sufficient accurate information about the contraceptive methods available to them, upon which they can base their decision-making (11). Challenges to all three of these principles were evidenced in our study: lack of access to implant removal services undermined women's full choice; provider resistance to performing early removals undermined women's free choice; poor pre-insertion counseling, including provision of information about the eventual removal procedure, undermined women's informed choice.

Provider resistance to performing removals has been noted in other settings where the implant is a novel method of contraception (27). This has been reported with current implant models, such as Implanon NXT® in South Africa (28), Ghana (18) and the United Kingdom (17), and with historic implant models, such as Norplant® in Indonesia (29) and Senegal (30). In our study, providers cited cost as a factor which dissuaded them from performing early removals because reducing the implant's lifespan decreases its cost-effectiveness. However, providers did not appear to weigh this financial expense against that associated with unintended pregnancies.

Our findings suggest that provider training needs to be reviewed to address deficiencies in pre-insertion counseling and improve support of women's right to choose early removal. Some users in our study chose to discontinue the implant due to lack of menstrual bleeding, and previous research has found a broad global variation in acceptability of bleeding changes caused by hormonal contraception (31). This highlights the need for context-specific, culturally-sensitive counseling throughout SRH services, including those for implant provision, particularly around expected side effects and how they can be managed. Providers can only offer a full choice of contraceptive options if the system they work in enables them to do so; therefore, management of the supply chain is needed in addition to individual provider training in order to facilitate women's full, free and informed contraceptive autonomy.

A key aim of our study was to elicit if personal experiences of the existing implant services were likely to impact upon wider perception of the implant as a method of contraception. Women who had encountered barriers to accessing implant removals were disappointed with this aspect of the service but remained positive about the method as a whole and said that they would recommend it to friends. Providers struck a more cautious note, expressing concerns that ongoing challenges in accessing implant removals would eventually damage the reputation of the implant and potentially reduce its uptake. This has already been witnessed in South Africa (28), where some users have resorted to removing their own implants (32). Women in our study had chosen to initiate the implant after hearing positive reviews from peers, so negative reviews might dissuade women from choosing the implant in the future. If access to implant removal services is not improved, a negative perception could develop amongst current users, potential users, and providers, which could lead to a decline in implant uptake.

Users' and providers' perceptions of contraceptive implants could also influence their future views about other types of implants, such as those currently in development for HIV prevention (33–35) and multipurpose prevention of both HIV and pregnancy (36). Given the high HIV prevalence in Botswana (25.1% among women of reproductive age) (37) and potential for these new implantable technologies to greatly reduce HIV incidence, it is crucial that current contraceptive implant services are optimized to pave the way for new types of medical implant.

## Limitations

This study has several limitations. It is small in size, and all users were recruited from and had undergone removal at one clinic. However, some users had tried to access removal services at six different clinics before eventually undergoing removal at the same clinic. The experiences of these users might not be representative of users who do successfully access implant removal services at other clinics. In the case of five users who presented directly to the NGO clinic for removal but who had originally inserted the implant elsewhere, we did not explore why they chose this clinic rather than attempt to obtain implant removal at the clinic of insertion. Both users and providers had experienced accessing and providing services in an urban setting, so their experiences might not be representative of implant services in rural areas. The users interviewed in this study were recruited opportunistically and had all sought early discontinuation of the implant; therefore, they may not be representative of the whole population of implant users, many of whom do not discontinue early. In common with other qualitative studies, there is also a risk of social desirability bias influencing participants' responses. Nevertheless, it is important to evaluate users' perspectives following the introduction of any new contraceptive method (38). Since the implant was introduced in Botswana in 2016, participants in our study represent users and providers who have been the first to experience Botswana's implant removal services.

## Conclusion

Upscaling provision of the contraceptive implant is an important strategy in increasing access to LARCs and promoting the reproductive health rights of women throughout low-resource settings. Expansion of implant services initially focused on insertions, while services for removals have lagged behind. Our study found that implant users and providers have experienced challenges in accessing and providing removals. For users, these challenges are principally in relation to an insufficient number of trained removal providers and provider resistance to performing early removals. For providers, the main challenges are an insufficient number of colleagues who are trained removal providers, insufficient materials, and lack of a referral pathway for difficult removals. It will be important to address these areas so that universal contraceptive access is achieved, including access to the means of method discontinuation. Access to removal services is a vital component in upholding women's contraceptive autonomy and maintaining user satisfaction with the implant. The findings of this study could help inform the ongoing development of services for implant insertion and removal in Botswana. We hope that other national SRH programs, particularly those operating in resource-limited settings, will benefit from learning about our experience.

## Data Availability Statement

The raw data supporting the conclusions of this article will be made available by the authors, without undue reservation.

## Ethics Statement

The studies involving human participants were reviewed and approved by the Botswana MoHW Health Research and Development Committee, the University of Botswana Research Ethics Committee, and University College London (UK) Research Ethics Committee. Permission was gained from the Botswana Family Welfare Association (BOFWA) and Princess Marina Hospital. The patients/participants provided their written informed consent to participate in this study.

## Author Contributions

CM and RH conceived the initial idea for the study. TK, SM, TM, and LM were involved in conceptualization of the study. RH, AG, and OB designed the protocol and study tools. AG, OB, and CB collected data. RH, AG, and OB conducted the analysis, with guidance from CM. RH drafted the initial manuscript and CM, EK, AM and DR-M contributed to subsequent drafts. All authors contributed to the article and approved the submitted version.

## Conflict of Interest

The authors declare that the research was conducted in the absence of any commercial or financial relationships that could be construed as a potential conflict of interest.

## Abbreviations

DMPA: depomedroxyprogesterone acetate
HIV: human immunodeficiency virus
IUD: intrauterine device
MoHW: Ministry of Health and Wellness
NGO: non-governmental organization
REDCap: Research Electronic Data Capture
SRH: sexual and reproductive health.
