# Supplementary material for: Experiences of Accessing and Providing Contraceptive Implant Removal Services in Gaborone, Botswana: A Qualitative Study Among Implant Users and Healthcare Providers
Source: Front Glob Womens Health. 2021 Jun 25;2:684694. doi: 10.3389/fgwh.2021.684694 (PMC8593984; doi:10.3389/fgwh.2021.684694)
Supplement: Supplementary file 1 [file Data_Sheet_1.PDF]

Supplementary Material

Supplementary Table 1: Key Codes and Sub-Codes Applied To User Interviews

**Codes**	**Sub-Codes**
1) Implant advantages	1a) Discreet<br>1b) Highly effective<br>1c) Long-acting<br>1d) No action required on part of user<br>1e) Rapid return to baseline fertility
2) Implant disadvantages	2a) Dislike of foreign object in body<br>2b) Invasiveness of insertion<br>2c) Invasiveness of removal<br>2d) Side effects
3) Positive experience of pre-removal	3a) Easy<br>3b) Kept updated<br>3c) No delay<br>3d) No interference with life<br>3e) No resistance from providers<br>3f) Removal information
4) Negative experience of pre-removal	4a) Delay<br>4b) Lack of trained providers<br>4c) Resistance from providers
5) Positive experience of removal	5a) Experienced provider<br>5b) Perception that providers are helpful
6) Negative experience of removal	6a) Interference with school/work<br>6b) Perception that providers are unhelpful
7) Post-removal contraception	7a) Switch to no contraception<br>7b) Switch to non-hormonal contraception<br>7c) Switch to hormonal contraception
8) Future implant use	8a) Unsure whether would use implant again<br>8b) Would consider using implant again<br>8c) Would not consider using implant again<br>8d) Would recommend implant to others

Supplementary Table 2: Key Codes and Sub-Codes Applied To Provider Interviews

**Codes**	**Sub-Codes**
1) Implant advantages	1a) National implant register<br>1b) No impact on bone density
2) Implant disadvantages	2a) Cannot self-discontinue<br>2b) Invasive procedure
3) No barriers to removal	3a) Equipment available<br>3b) Other clinics available
4) Barriers to removal	4a) Cost of implant<br>4b) Doctors unable to provide removal<br>4c) Insufficient equipment<br>4d) Insufficient time<br>4e) Lack of trained providers<br>4f) Multiple clinic visits<br>4g) No referral pathway for difficult removals<br>4h) Removal difficult
5) Sufficient training	5a) Feel competent after basic training<br>5b) Feel competent after extra training<br>5c) Sufficient duration<br>5d) Sufficient mentoring<br>5e) Sufficient on insertion<br>5f) Sufficient on side effects<br>5g) Sufficient on standard removals<br>5h) Sufficient practical experience
6) Insufficient training	6a) Do not feel competent after basic training<br>6b) Insufficient duration<br>6c) Insufficient mentoring<br>6d) Insufficient on difficult removals<br>6e) Insufficient on insertion<br>6f) Insufficient on Jadelle®<br>6g) Insufficient on side effects<br>6h) Insufficient on standard removal<br>6i) Insufficient practical experience
7) Perception of implant	7a) Impact of removal services on reputation of implant<br>7b) Would not use implant personally<br>7c) Would recommend implant promotion in Botswana<br>7d) Would recommend implant to a family member<br>7e) Would use implant personally
